# Supplementary material for: The role of Hurst exponent on cold field electron emission from conducting materials: from electric field distribution to Fowler-Nordheim plots
Source: Sci Rep. 2015 Jun 2;5:10175. doi: 10.1038/srep10175 (PMC4451801; doi:10.1038/srep10175)
Supplement: Supplementary Information [file srep10175-s1.pdf]

The role of Hurst exponent on cold field electron emission from conducting materials: from electric field distribution to Fowler-Nordheim plots

T. A. de Assis[1,2]

[1]*Instituto de Física, Universidade Federal da Bahia, Campus Universitário da Federação, Rua Barão de Jeremoabo s/n, 40170-115, Salvador, BA, Brazil*
[2]*Instituto de Física, Universidade Federal Fluminense, Avenida Litorânea s/n, 24210-340 Niterói RJ, Brazil*[*]

[*] thiagoaa@ufba.br

Supplementary Information

Numerical method for electric field calculations

Assuming that the region between the emitter surface and the anode is vacuum, this implies the numerical solution of Laplace's equation,

$$\nabla^2\Phi = 0, \tag{1}$$

in a discretized space, imposing suitable boundary conditions. In our case, they correspond to Dirichlet conditions both at the cathode ($\Phi = 0$) and the anode ($\Phi = \Phi^{\mathrm{A}}$), and periodic lateral conditions, so that for a domain formed by $L_x \times L_y \times L_z$ points (in the directions x, y, z, respectively), we have

$$\Phi_{i,j,k} = \Phi_{i+L_x,j,k} = \Phi_{i,j+L_y,k} = \Phi_{i+L_x,j+L_y,k}. \tag{2}$$

Equation (1) is then solved iteratively using a second order finite difference scheme, in which the electric potential is given by

$$\Phi^{(m+1)}_{i,j,k} = \frac{1}{6}[\Phi^{(m)}_{i-\omega,j,k} + \Phi^{(m)}_{i+\omega,j,k} + \Phi^{(m)}_{i,j-\omega,k} + \Phi^{(m)}_{i,j+\omega,k} + \Phi^{(m)}_{i,j,k-\omega} + \Phi^{(m)}_{i,j,k+\omega}]. \tag{3}$$

That is, at each iteration the new potential $\Phi^{(m+1)}_{i,j,k}$ is just the average of its magnitude on the surrounding points at the previous iteration, (m). These values are then refined iteratively starting from the first step, $\Phi^{(0)}_{i,j,k} = 0$, until a satisfactory convergence criterion

$$\epsilon_{\max} = \max\{\epsilon_{i,j,k}\} < \xi, \tag{4}$$

where

$$\epsilon_{i,j,k} = \left| \frac{\Phi^{(m+1)}_{i,j,k} - \Phi^{(m)}_{i,j,k}}{\Phi^{(m+1)}_{i,j,k}} \right|, \tag{5}$$

is met. In this work, the error is set to be $\xi = 10^{-6}$. In Eq.(3), ω is the length of lattice parameter in the cubic lattice. In this work, we refine the grid in such that $\omega = 1/50u$, where u is the basic unit distance. However, no significatively deviations has been detected using $\omega = u$. Once convergence has been achieved, the intensity of the electric field,

$$\mathbf{F}(\mathbf{r}) = -\nabla\Phi(\mathbf{r}), \tag{6}$$

at a any point (not necessarily on the grid), $\mathbf{r}_P = (x_P, y_P, z_P)$, can be evaluated by linear interpolation from the values at the eight vertices of the circumscribing grid cube. If such a cube "starts" at the grid location (i, j, k), the corresponding cartesian components, $F^{x,y,z}(\mathbf{r})$, are given by

$$F^{\mu}(r) = N \sum_{\alpha_i,\alpha_j,\alpha_k=0}^{\omega} \frac{E^{\mu}_{i+\alpha_i,j+\alpha_j,k+\alpha_k}}{r(x_{P,\alpha_i}, y_{P,\alpha_j}, z_{P,\alpha_k})}, \tag{7}$$

where:

$$\frac{1}{N} = \sum_{\alpha_i,\alpha_j,\alpha_k=0}^{\omega} \frac{1}{r(x_{P,\alpha_i}, y_{P,\alpha_j}, z_{P,\alpha_k})}, \tag{8}$$

and

$$r(x_{P,\alpha_i}, y_{P,\alpha_j}, z_{P,\alpha_k}) \equiv \sqrt{(x_P - x_{i+\alpha_i})^2 + (y_P - y_{j+\alpha_j})^2 + (z_P - z_{k+\alpha_k})^2}. \tag{9}$$

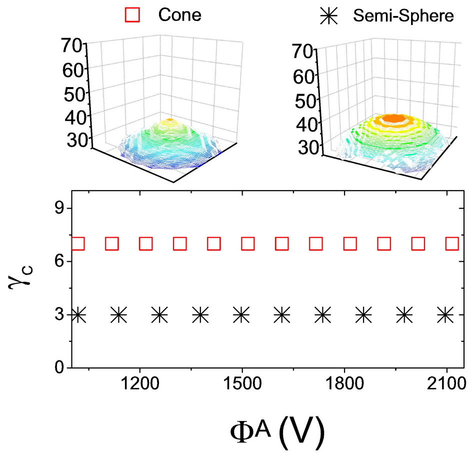

FIG. S1 - Characteristic FEF, evaluated numerically by the solution of the Laplace equation, as a function of the anode electric potential, Φ^A, considering a semi-sphere and a cone protuberances superimposed to a planar conducting surface.

To test the accuracy of our method, we consider calculate the characteristic FEF, γ_C, solving the electric field in three dimensions around a conducting sphere (and conical) structure superimposed in the center of a planar conducting substrate. The electric potential at the surface of the conducting sphere was fixed to $\Phi = 0$, while at the anode a electric potential Φ^A. The results for γ_C of the two structures are presented in Fig. S1, for $\Delta x_i, \Delta x_j \ll L$, as a function of the electric potential Φ^A. It's clear the very good agreement between our numerical results and the analytical solution which predicts $\gamma_C = 3$ for the semi-sphere on a plane [1]. In the case of conical structure, our results are in accordance with those presented on reference [2].

Considerations regarding two-dimensional electric field variation across the LACFES

In this Supplementary Information section, we wish to demonstrate that the same relevant results obtained in the case of the one-dimensional electric field variation across the LACFES (using orthodox cold field electron emission theory) are also obtained for a genuine rough three-dimensional LACFES setup (where a two-dimensional electric field variation across the LACFES is considered). For this purpose, we use fractional Brownian motion (fBm) algorithms [3] to generate random self-affine objects with specific Hurst exponents H. FBm algorithms can be generalized to higher dimensions using a multidimensional process such that for three dimensions, an irregular LACFES of height $h(x,y)$ results in interfaces that are statistically equivalent in all directions. We consider the self-affine sets that are constructed using the well-known midpoint displacement algorithm for H=0.1 [4].

In Fig. S2, we illustrate the rough LACFESs produced using the described fBm algorithm for a Hurst exponent of H=0.1 and with global roughnesses $W \approx 20$ nm, 37.5 nm and 0.5μm. In Fig. S3 (top), several equipotential surfaces are shown for the case in which the electric potentials of the LACFES (with $H = 0.1$ and $W \approx 37.5$ nm) and the far-away conducting anode are $\Phi^S = 0$ V and $\Phi^A = 500$ V, respectively, corresponding to Dirichlet conditions. We apply this approximation, following the same procedure used in the one-dimensional electric field variation across the

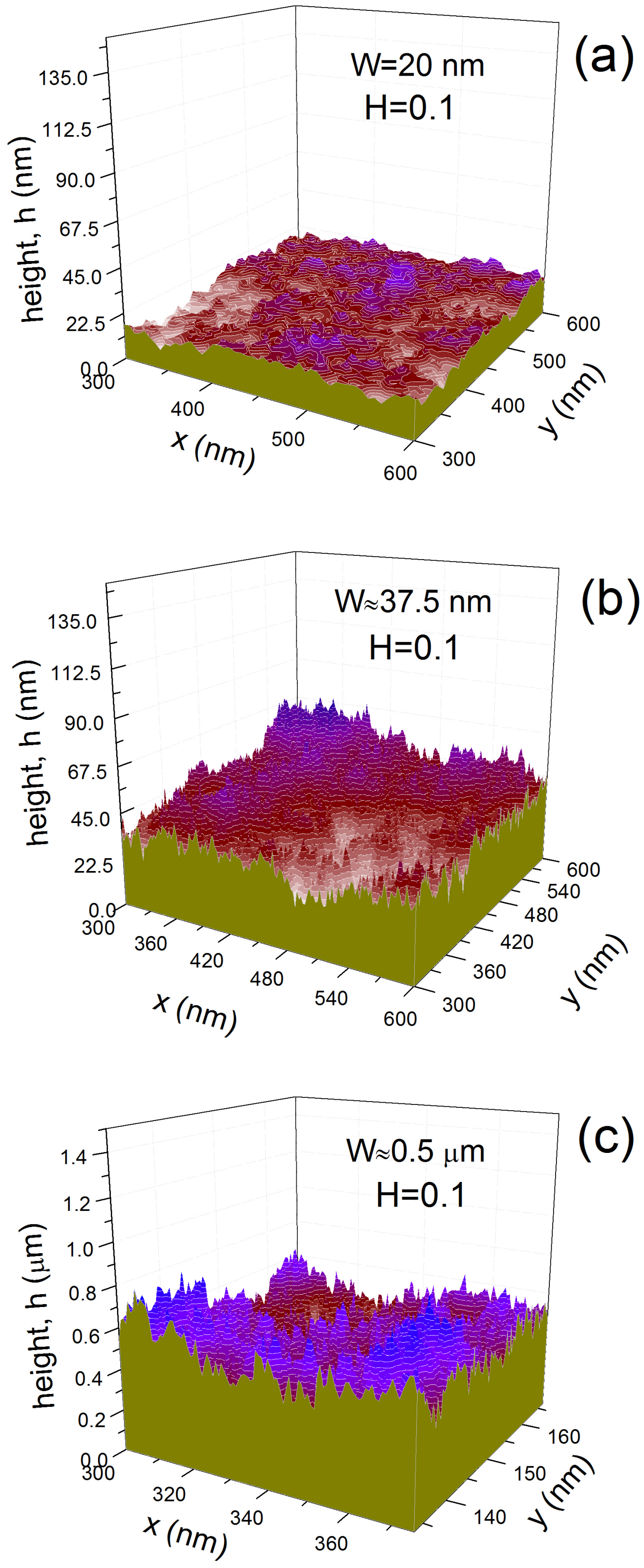

FIG. S2 - Portion of rough LACFES generated using the fBm algorithm for a Hurst exponent of H=0.1 and global roughnesses: (a) $W \approx 20$ nm; (b) $W \approx 37.5$ nm and (c) $W \approx 0.5\mu$m.

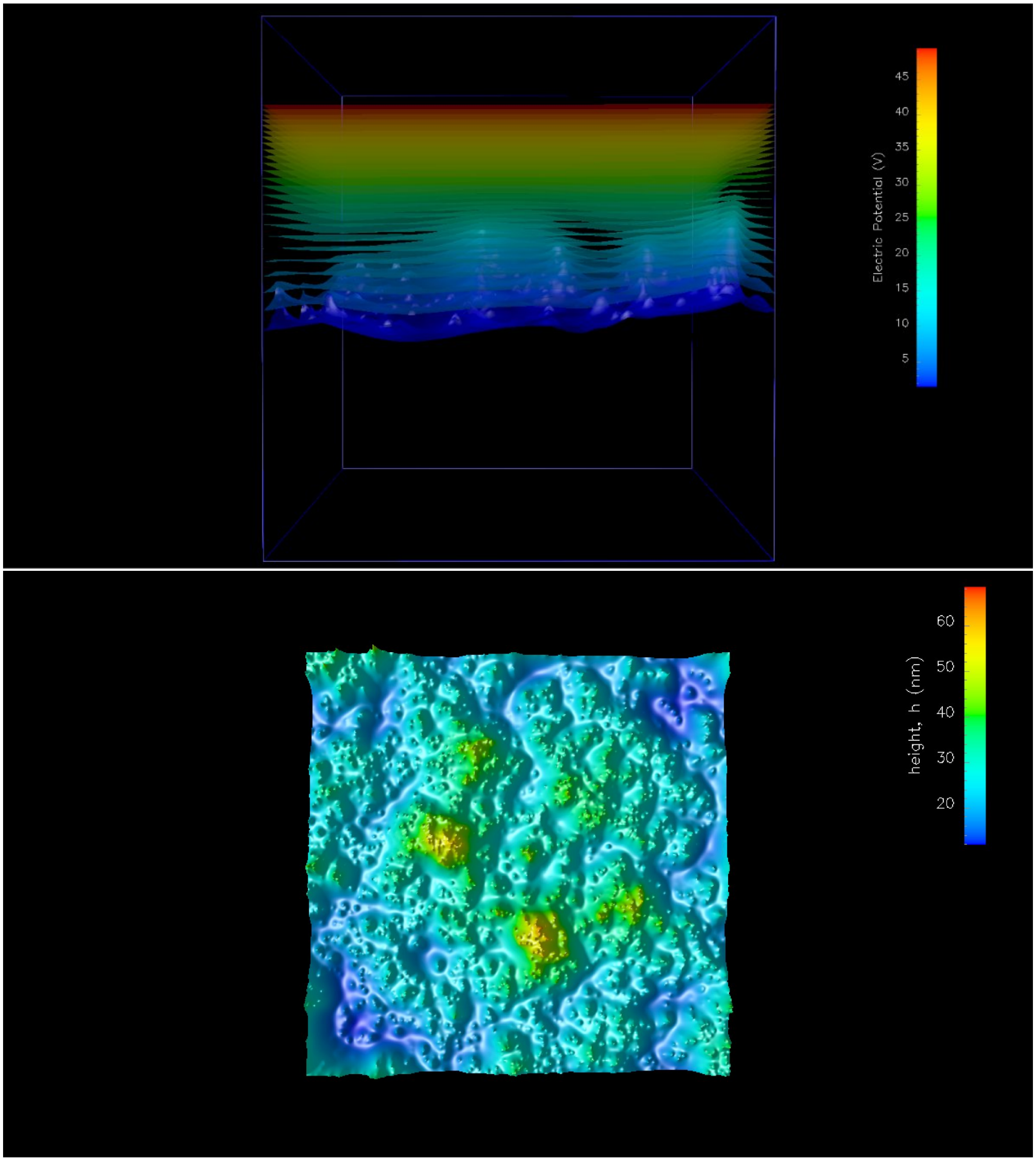

FIG. S3 - (top) Snapshot showing several equipotential surfaces calculated numerically from the solution of the Laplace equation, using the LACFES represented in Fig. S2(b) with appropriate Dirichlet conditions ($\Phi^S = 0$ V and $\Phi^A = 500$ V). The color bars indicate the electric potential values (in Volts). (bottom) The equipotential surface that is used as an approximation to the field emitter surface corresponding to the real fBm surface, defined by $\Phi^E = 1$ V. The color bars indicate the height of LACFES (in nanometers).

LACFES, with the intent of rounding the sharpest projections. In this way, we replace the real fBm surface with an equipotential surface, $\Phi^E = 1$ V (very similar to the previous one), that represents an approximation of the field emitter surface (see Fig. S3 (bottom)). Again, following the same procedure using the WM function, this methodology ensures that on small scales (though larger than the atomic scale), the local roughness is negligible, whereas on large scales (though much less than the lateral size of the system, L), the local roughness scales with the same exponent H.

In Fig. S4, we present the local FEF distributions, $\rho(\gamma)$, for LACFESs shown in Fig. S2. Interestingly, exponential behaviors (two exponentially decaying regions as well as a characteristic FEF factor of the same order, for $W \approx 37.5$ nm and $W \approx 0.5\mu$m, as that observed in thin-film emitters with irregular surfaces - see Ref.[30] of the manuscript) are observed in all cases, the same behavior as that discussed in the case of WM surfaces, when the one-dimensional

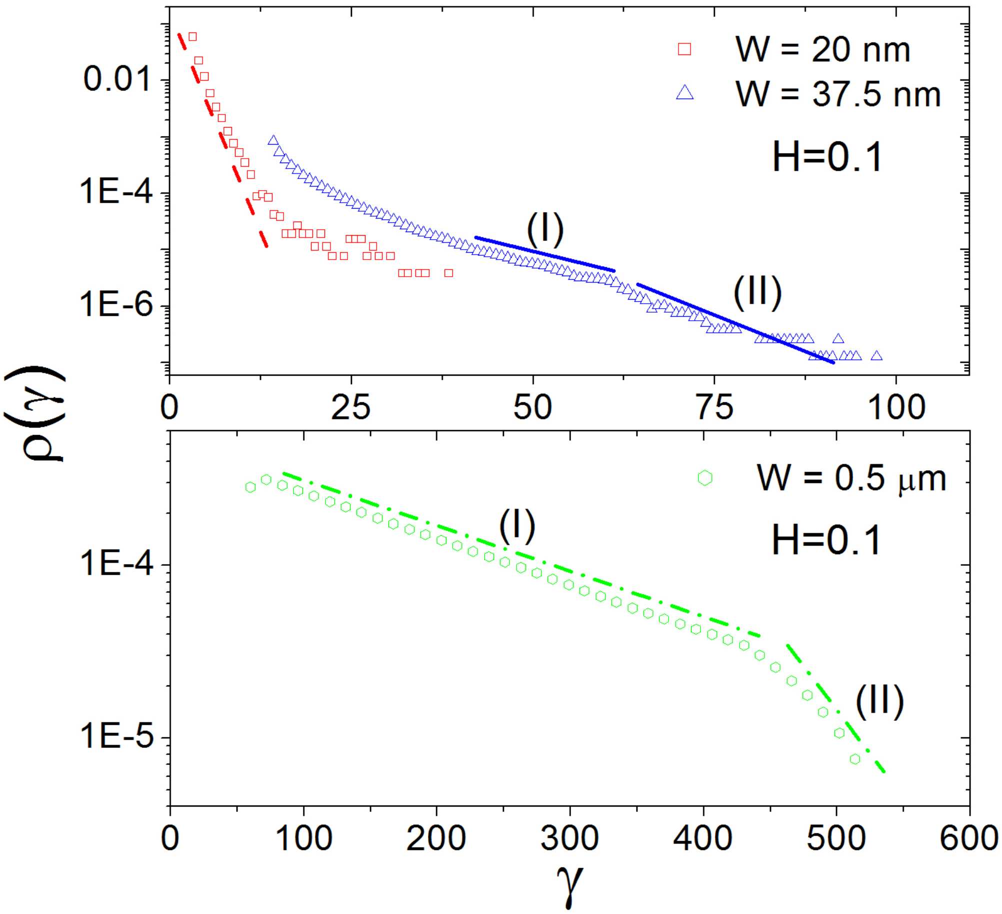

FIG. S4 - Local FEF distributions, $\rho(\gamma)$, for LACFESs constructed using the fBm algorithm for $H = 0.1$ with global roughnesses of $W \approx 20$ nm (red squares), $W \approx 37.5$ nm (blue triangles) and $W \approx 0.5\mu$m (green hexagons). Exponentially decaying behavior can be clearly observed in all cases. In particular, for $W \approx 37.5$ nm and $W \approx 0.5\mu$m, the same behavior previously observed, that of two exponentially decaying regimes, is apparent. The slope of the dashed (red) line is (-0.247 ± 0.008). The slopes of the solid (blue) lines are (-0.0324 ± 0.0001) (region (I)) and (-0.046 ± 0.001) (region (II)). The slopes of the dot-dashed (green) lines are (-0.00273 ± 0.00005) (region (I)) and (0.0090 ± 0.0001) (region (II)) (see Table SI). The differences observed in the $\rho(\gamma)$ distributions are essentially related to the difference in global roughness between the LACFESs.

local electric field variation across the emitter profile is considered. Thus, the one-dimensional case is an interesting tool that captures the main results experimentally obtained for rough LACFESs, as noted previously.

Note that for $H = 0.1$ and $W \approx 37.5$ nm [$\approx 0.5\mu$m] (in which case the characteristic FEF has been found to be $\gamma_C \approx 97.24$ [≈ 519.74]), two exponentially decaying regimes are evident, labeled as (I) and (II), which correspond to $\rho^I(\gamma) \sim \exp(-\delta_H^I \gamma)$ and $\rho^{II}(\gamma) \sim \exp(-\delta_H^{II} \gamma)$, respectively, where $\delta_H^{II} > \delta_H^I$. For $W \approx 37.5$ [$\approx 0.5\mu$m], $\delta_{0.1}^I \times \log_{10}(e) = (0.0324 \pm 0.0001)$ [$\delta_{0.1}^I \times \log_{10}(e) = (0.00273 \pm 0.00005)$] is identified for interval (I), and $\delta_{0.1}^{II} \times \log_{10}(e) = (0.046 \pm 0.001)$ [$\delta_{0.1}^{II} \times \log_{10}(e) = (0.0090 \pm 0.0001)$] is found for interval (II). By contrast, for $W \approx 20$ nm, only one exponentially decaying regime is observed, with $\delta_{0.1}^I \times \log_{10}(e) = (0.247 \pm 0.008)$. This finding indicates that the $\rho(\gamma)$ distribution is also sensitive to changes in the global roughness of the LACFES. Differences between the theoretically obtained values of the characteristic FEF and those obtained experimentally can also be caused by differences in the global roughness. Table SI summarizes the values of the parameters extracted from the $\rho(\gamma)$ distributions presented in Fig. S4 and the γ_C values for all values of W explored.

Finally, we show that the β_W (note that the notation is now used with W for global roughness) correction, which includes the effect of the geometry of the real three-dimensional LACFES setup on the estimation of the characteristic FEF, must be considered to ensure more precise estimation of γ_C (which was found to be $\gamma_C \approx 97.24$ [≈ 519.74] from the $\rho(\gamma)$ distribution depicted in Fig. S4 for $W \approx 37.5$ nm [$\approx 0.5\mu$m]). We restrict this discussion to $H = 0.1$ and for roughnesses $W \approx 37.5$ nm and $W \approx 0.5\mu$m, i.e., to the LACFESs represented in Figs. S2 (b) and (c), respectively.

Fig. S5(a), shows the behavior of the corresponding J_M-F_M-type FN plots. The FN plots seems, again, to exhibit approximately linear behavior for the considered range of macroscopic electric field F_M, although, in reality, the

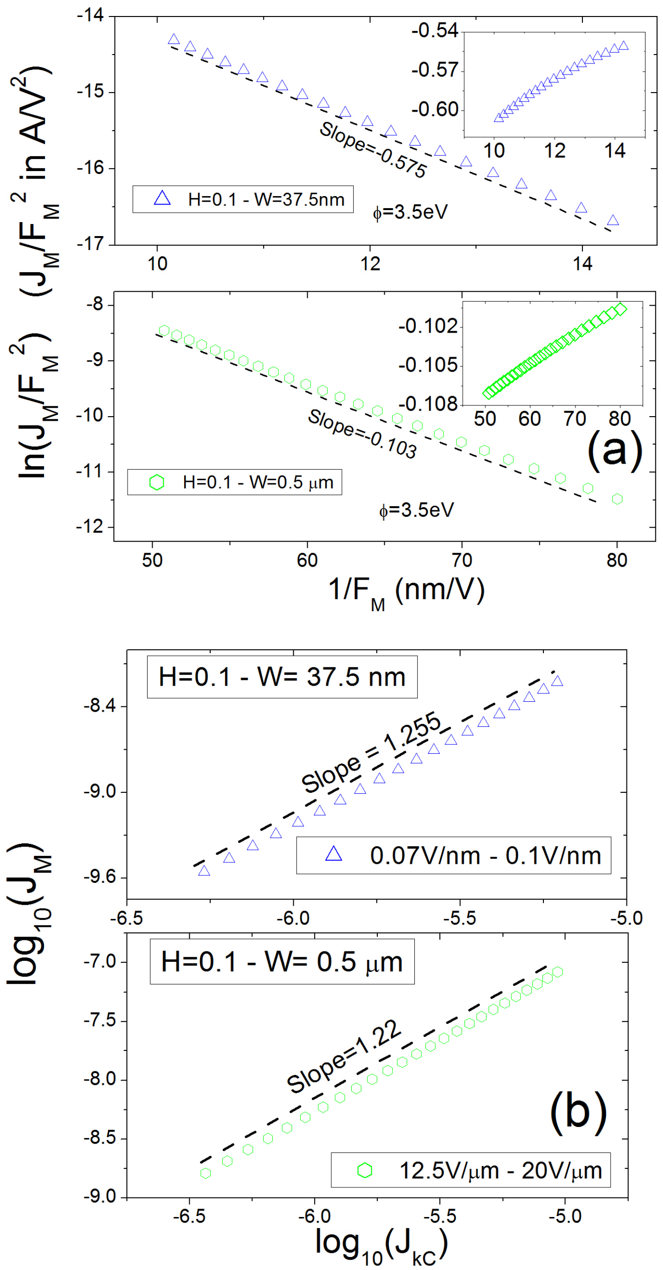

FIG. S5 - (a) J_M-F_M-type FN plot for LACFESs with $H = 0.1$ and $W \approx 37.5$ nm (blue triangles) and $W \approx 0.5\mu$m (green hexagons). The corresponding slopes (dashed lines) are shown (see Table SII). The work function of the LACFES is considered to be approximately constant and equal to $\phi = 3.5$ eV. The inset of this figure shows the corresponding derivatives of the J_M-F_M-type FN plots for LACFESs. (b) Macroscopic current density, J_M as a function of the characteristic kernel current density, J_{kC} with $H = 0.1$ and $W \approx 37.5$ nm (blue triangles) and $W \approx 0.5\mu$m (green hexagons). For $W \approx 37.5$ nm and $W \approx 0.5\mu$m the macroscopic electric fields are in the range $0.07V/nm \leq F_M \leq 0.1V/nm$ and $12.5V/\mu m \leq F_M \leq 20V/\mu$m, respectively. The slopes (values of β_W) are also indicated (see Table SII).

derivative is not constant, as observed in the corresponding inset.

Fig. S5(b) shows the behavior of $\log_{10}(J_M)$ as a function of $\log_{10}(J_{kC})$, considering a macroscopic electric field in the range $0.07V/nm \leq F_M \leq 0.1V/nm$ and $12.5V/\mu m \leq F_M \leq 20V/\mu m$ for $W \approx 37.5$ nm and $W \approx 0.5\mu$m, respectively. These results suggest, again, a scaling relation between J_M and J_{kC} with $\beta_W = (1.255 \pm 0.001)$ for $W \approx 37.5$ nm [and $\beta_W = (1.222 \pm 0.002)$ for $W \approx 0.5\mu$m]. From these results, for $W \approx 37.5$ nm [$\approx 0.5\mu$m] we calculate an elementary slope characterization parameter of $\gamma_C^T \approx 77.7$ [≈ 434] (error of 21% [16.4 %] with respect to the γ_C value found from the $\rho(\gamma)$ distribution). If we include the corrections σ (which is a generalized slope correction factor related to the SN barrier) and β_W (which includes the effect of the global roughness of the LACFES on the

W	$\delta_H^I \times \log_{10}(e)$	$\delta_H^{II} \times \log_{10}(e)$	γ_C
≈ 20 nm	0.247 ± 0.008	–	≈ 38.35
≈ 37.5 nm	0.0324 ± 0.0001	0.046 ± 0.001	≈ 97.24
$\approx 0.5\mu$m	0.00273 ± 0.00005	0.0090 ± 0.0001	≈ 519.74

TABLE SI - Results of the extraction of parameters from the $\rho(\gamma)$ distribution shown on Fig. S4 for $H = 0.1$ and global roughnesses $W \approx 20$ nm, 37.5 nm and 0.5 μm. For $W \approx 20$ nm a dominant exponential decay can be observed in the $\rho(\gamma)$ distribution, characterized by δ_H^I.

W	$S_{F_M}(W)(V/nm)$	γ_C^T	β_W	$\gamma_C^{\beta_W,\sigma}$
≈ 37.5 nm	-0.5759 ± 0.0009	77.77 ± 0.01	1.255 ± 0.001	93.2 ± 0.3
$\approx 0.5\mu$m	-0.1031 ± 0.002	434.2 ± 0.1	1.224 ± 0.001	505.88 ± 0.01

TABLE SII - Results for $H = 0.1$ and $W \approx 20$ nm, 37.5 nm and 0.5μm. The slopes of the J_M-F_M-type FN plots shown on Fig. S5(a), characteristic FEF γ_C^T, the β_W values extracted from the results presented on Fig. S5(b) and $\gamma_C^{\beta_W,\sigma}$. γ_C^T and $\gamma_C^{\beta_W,\sigma}$ were calculated using Eqs. (21) and (23) of the manuscript while considering the elementary FN equation and relevant corrections, including the SN barrier and the morphology of the LACFES, respectively.

estimation of the characteristic FEF), then the corrected slope characterization parameter is found to be $\gamma_C^{\beta_W,\sigma} \approx 93$ [≈ 505.8], yielding an error of 4% [2.6%] with respect to the γ_C value determined from the $\rho(\gamma)$ distribution (see Table SII for values of the slopes of the J_M-F_M-type FN plots shown in Fig. S5(a), the characteristic FEF values γ_C^T, the β_W values extracted from the results presented in Fig. S5(b) and $\gamma_C^{\beta_W,\sigma}$).

In conclusion, the scale that results in the exponent β_W must be considered (including in the case of a two-dimensional electric field variation across the LACFES and in addition to the generalized slope correction from the SN barrier) in the calculation of the slope characterization parameter that is used by experimentalists to extract a more precise characteristic FEF, such as that found using the one-dimensional WM function.

SUPPLEMENTARY REFERENCES

[1] Forbes, R. G., Edgcombeb, C.J. & Valdrè, U., Some comments on models for field enhancement. *Ultramicroscopy* **95**, 57-65 (2003).

[2] Mustonen, A., Beaud, P., Kirk, E., Feurer, T. & Tsujino, S., Efficient light coupling for optically excited high-density metallic nanotip arrays. *Sci. Rep.* **2**, 915.1-915.5 (2012).

[3] Feder, J., Fractals *Plenum Press, New York* (1988).

[4] Kikkinides, E. S. & Burganos V. N., Permeation properties of three-dimensional self-affine reconstructions of porous materials. *Phys. Rev. E* **62**, 6906-6915 (2000).
